# Supplementary data on rapid sample clean-up procedure of aminophosphonates for LC/MS analysis

**DOI:** 10.1016/j.mex.2020.100933

**Published:** 2020-05-23

**Authors:** Ramona Kuhn, Isaac Mbir Bryant, Marion Martienssen

**Affiliations:** Brandenburg University of Technology Cottbus-Senftenberg, Institute of Environmental Technology, Chair of Biotechnology of Water Treatment, 03046 Cottbus, Germany

**Keywords:** Phosphonates, LC/MS analysis, Cation Exchange Resin, Clean-up

## Abstract

Minimising matrix effects through high sample purity is of major importance for LC/MS analysis. Here we provide supplementary data and protocols related to the article “Rapid sample clean-up procedure of aminophosphonates for LC/MS analysis” (revised article submitted to Talanta) [1]. It is demonstrated that the tested phosphonates iminodi(methylenephosphonic acid) (IDMP), hydroxyethelidene(diphosphonic acid) (HEDP), aminotris(methylenephosphonic acid) (ATMP), ethylenediaminetetra(methyloenephosphonic acid) (EDTMP) and diethylenetriaminepenta(methylenephosphonic acid) (DTPMP) dissolved in tap water are not detectable by LC/MS without sample clean-up. Only the smallest aminophosphonate amino(methylenephosphonic acid) (AMPA) was detectable but the recovery is decreased drastically.

The optimised sample clean-up with cation exchange resin (CER) Dowex 50WX8 is described in detail and illustrated. The protocol is provided. The influence of the incubation time, addition of different ammonium acetate concentrations, different samples pHs and different water qualities is demonstrated and preferred clean-up conditions are recommended. Calibration results of all tested aminophosphonates are validated regarding limit of detection, limit of quantification, lower limit of quantification, absolute and relative process standard deviation. A final recommendation for the best clean-up condition for all six tested aminophosphonates is provided.

• *AMPA analysis without derivatisation is possible with optimised clean-up procedure*
• *Clean-up procedure is combinable with derivatisation method of* [2]
• *Procedure is simple, rapid and highly reproducible*

## Graphical Abstract

**Figure:**
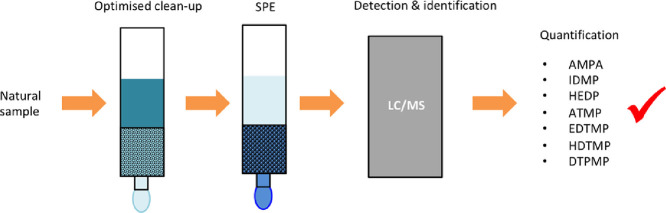

Specifications Table

**Table:** 

Subject Area	• Chemistry
More specific subject area:	*Sample clean-up of highly polar substances for LC/MS analysis*
Method name:	*Sample clean-up applying strong cation exchange resin Dowex 50WX8*
Name and reference of original method	S. Wang, S. Sun, C. Shan, B. Pan, Analysis of trace phosphonates in authentic water samples by pre-methylation and LC-Orbitrap MS/MS, Water Res. 161 (2019) 78-88. https://doi.org/10.1016/j.watres.2019.05.099. *C.K. Schmidt, B. Raue, H.J. Brauch, F. Sacher, Trace-level analysis of phosphonates in environmental waters by ion chromatography and inductively coupled plasma mass spectrometry, Intern. J. Environ. Anal. Chem. 94 (2014) 385-398*, https://doi.org/10.1080/03067319.2013.831410.
Resource availability	*NA*

## Background

Sample analysis of phosphonates based on LC-ESI-MS allows precise quantification and identification of known and unknown phosphonate structures. Commonly, solid-phase extraction (SPE) has been used to pre-concentrate and clean-up liquid samples by minimising matrix effects, enriching and purifying traces of environmental samples for LC/MS analysis [3]. However, samples containing phosphonates require more specific purification, especially, if higher concentrations of cations are expected. Those obstacles can start rising with simple surface water or TW samples containing anions and cations in higher concentrations compared to purified water for analysis. First attempts to purify and pre-concentrate samples containing phosphonates were recently reported [4]. Different exchange resins were tested but resulted in only low recoveries. Also liquid-liquid extraction was not efficient. According to Schmidt et al. [5] anions such as sulphate and chloride from water samples can be discharged during the chromatographic run by including a switching step between the pre-column and the main chromatographic column. The removal of cations seems to be a greater challenge since phosphonates form metal complexes. Therefore, Schmidt et al. [5] recommended additional sample purification with cation exchange resin (CER).

The developed sample clean-up refers to Schmidt et al. [5] and also to Wang et al. [2] who both applied CER purification of phosphonates prior to LC/MS analysis. In both cases amino(methylenephosphonic acid) (AMPA) was not determinable due to strong adsorption on the resin. We investigated this phenomenon more in detail and describe here the specific clean-up protocol applying strong cation exchange resin (CER) Dowex 50WX8 for AMPA and other common aminophosphonates. The samples are analysable by LC/MS without derivatisation.

## Phosphonates

**Fig. 1:**
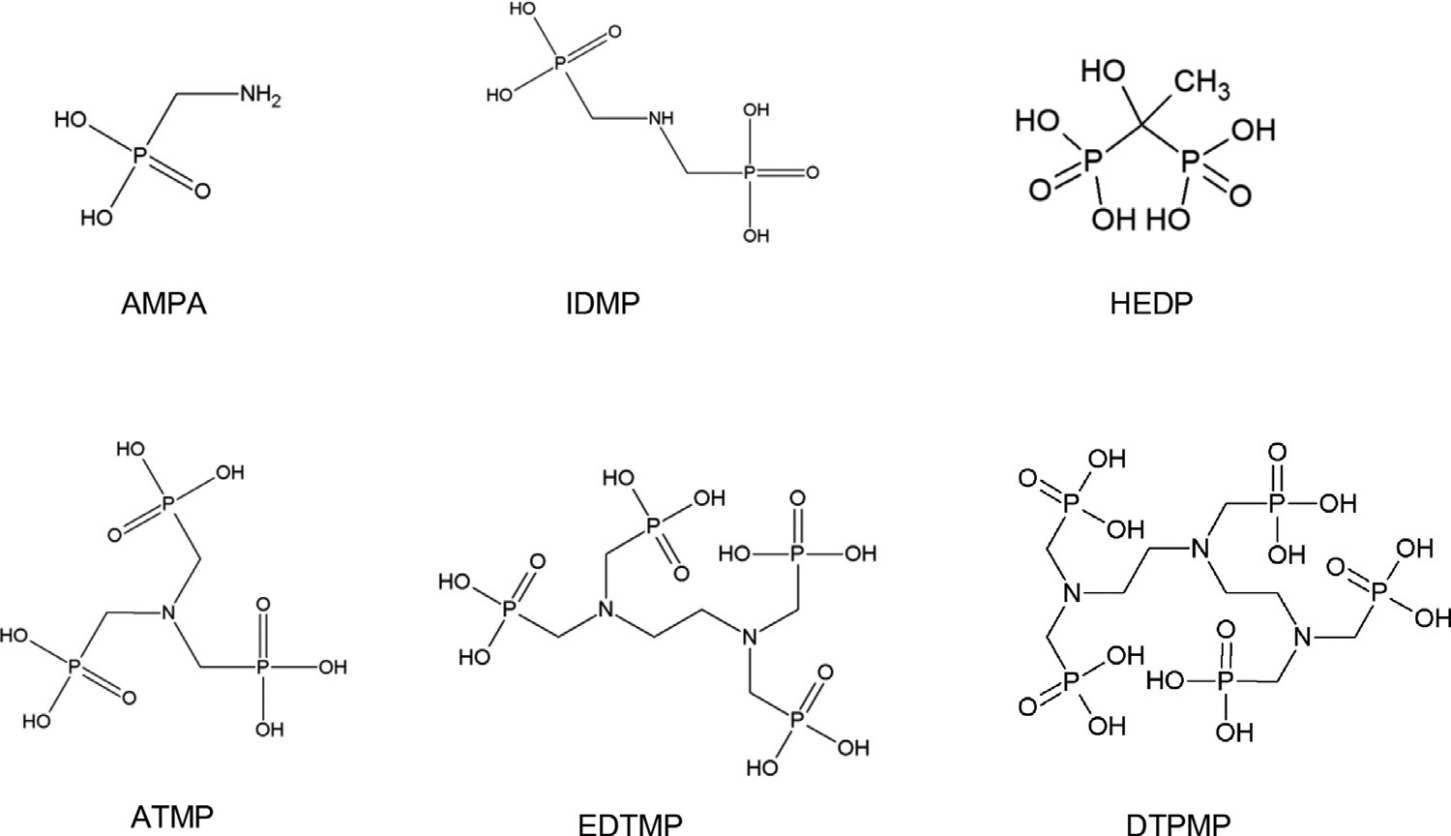
Chemical structure of tested phosphonates.

The six standard phosphonates AMPA, iminodi(methylenephosphonic acid) (IDMP), hydroxyethelidene(diphosphonic acid) (HEDP), aminotris(methylenephosphonic acid) (ATMP), ethylenediaminetetra(methyloenephosphonic acid) (EDTMP) and diethylenetriaminepenta(methylenephosphonic acid) (DTPMP) were used for the method development. All standards were of analytical grade or better with purity > 99 %. The chemical structures are presented below (Fig. 1).

## Standard solutions

1. Standard mixture stock solution in ultra pure water (UPW): 500 mg L^−1^ of each phosphonate (i.e. AMPA, IDMP, HEDP, ATMP, EDTMP and DTPMP)
2. Standard solutions in UPW: 100 mg L^−1^, 50 mg L^−1^, 25 mg L^−1^, 10 mg L^−1^ and 5 mg L^−1^ of each phosphonate (i.e. AMPA, IDMP, HEDP, ATMP, EDTMP and DTPMP) is achieved by diluting the UPW mixture stock solution
3. Standard mixture stock solution in tap water (TW): 500 mg L^−1^ of each phosphonate (i.e. AMPA, IDMP, HEDP, ATMP, EDTMP and DTPMP)
4. Standard solutions in TW: 100 mg L^−1^, 50 mg L^−1^, 25 mg L^−1^, 10 mg L^−1^ and 5 mg L^−1^ of each phosphonate (i.e. AMPA, IDMP, HEDP, ATMP, EDTMP and DTPMP) is achieved by diluting the TW mixture stock solution

All solutions can be stored at 4°C in the dark for at least one week. Optional different ammonium acetate (CH_3_COONH_4_) concentrations can be adjusted if required. For the optimised clean-up procedure either 100 mg L^−1^ or 1000 mg L^−1^ CH_3_COONH_4_ were added to the standard solutions (see section method validation). Standard solutions and samples prepared in waters with very low cation concentrations, e.g. in UWP, may require more addition of CH_3_COONH_4_ (commonly between 1000 mg L^−1^ and 4000 mg L^−1^). Standard solutions and samples prepared in waters with higher cation concentrations, e.g. in TW, fresh water or surface water, may require less addition of CH_3_COONH_4_ (commonly between 100 mg L^−1^ and 500 mg L^−1^).

## Materials

1. Dowex 50WX8 with 100-200 mesh (hydrogen form; Acros Organics, Geel, Belgium)
2. Vacuum filtration unit (VacMaster sample processin station, Biotage, Sweden)
3. Bond elution reservoirs (inner diameter 1.27 cm, Agilent Technologies, USA)
4. Frits (1/2 inch; Agilent Technologies, USA)
5. LC column (ZIC HILIC; Merck, Germany)

## Analytical instrumentation

Purified samples can be either analysed by ion chromatography (IC), high pressure liquid chromatography (HPLC) or LC-ESI-MS/MS. For HPLC analyses derivatisation might be favoured. For LC/MS analyses samples can be used without or with derivatisation depending on the instrumental set-up available. In this study, purified samples were analysed without derivatisation according to the LC/MS method recently published [1].

## Protocol

### Conventional sample clean-up procedure with Dowex 50WX8

**Fig. 2:**
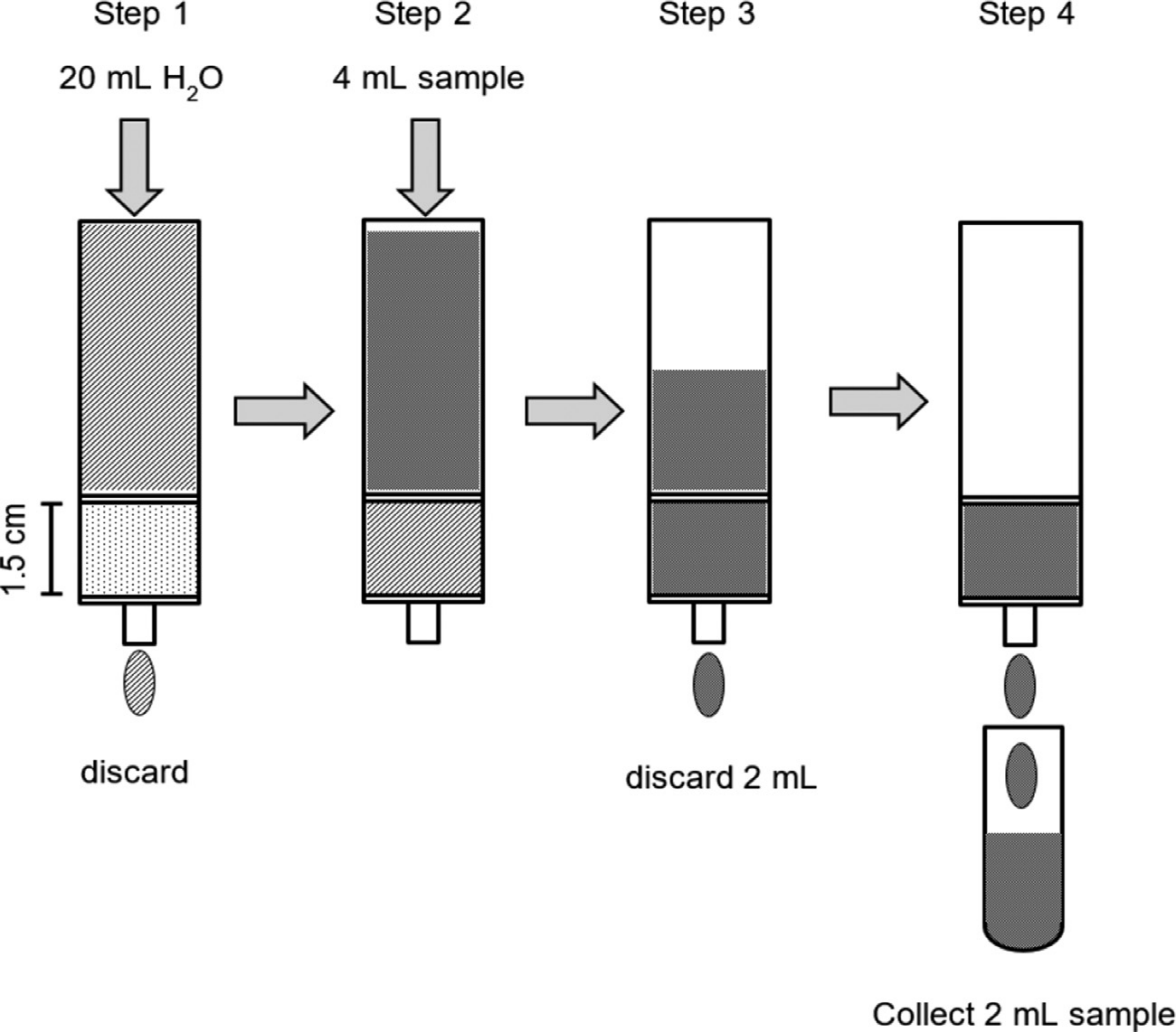
Scheme of the conventional sample clean-up procedure with Dowex 50WX8.

The complete procedure is illustrated below (Fig. 2).

1. Set 6 mL bond elution reservoir in vacuum filtration unit
2. Fit reservoir with frit at the bottom
3. Fill up the reservoir with homogenised wet Dowex 50WX8 corresponding to 1.75 g wet resin material
4. Cover resin with frit
5. Rinse CER bed with 20 mL UPW (step 1)
6. Load 4 mL standard sample solution containing phosphonates such as AMPA (step 2)
7. Elute standard solution with a flow rate of 1 mL min^−1^ and discard the initial 2 mL of the eluent (step 3)
8. Collect the final 2 mL of the eluent (step 4)
9. Use purified sample for analysis

### Optimised sample clean-up procedure with Dowex 50WX8 for aminophosphonates

**Fig. 3:**
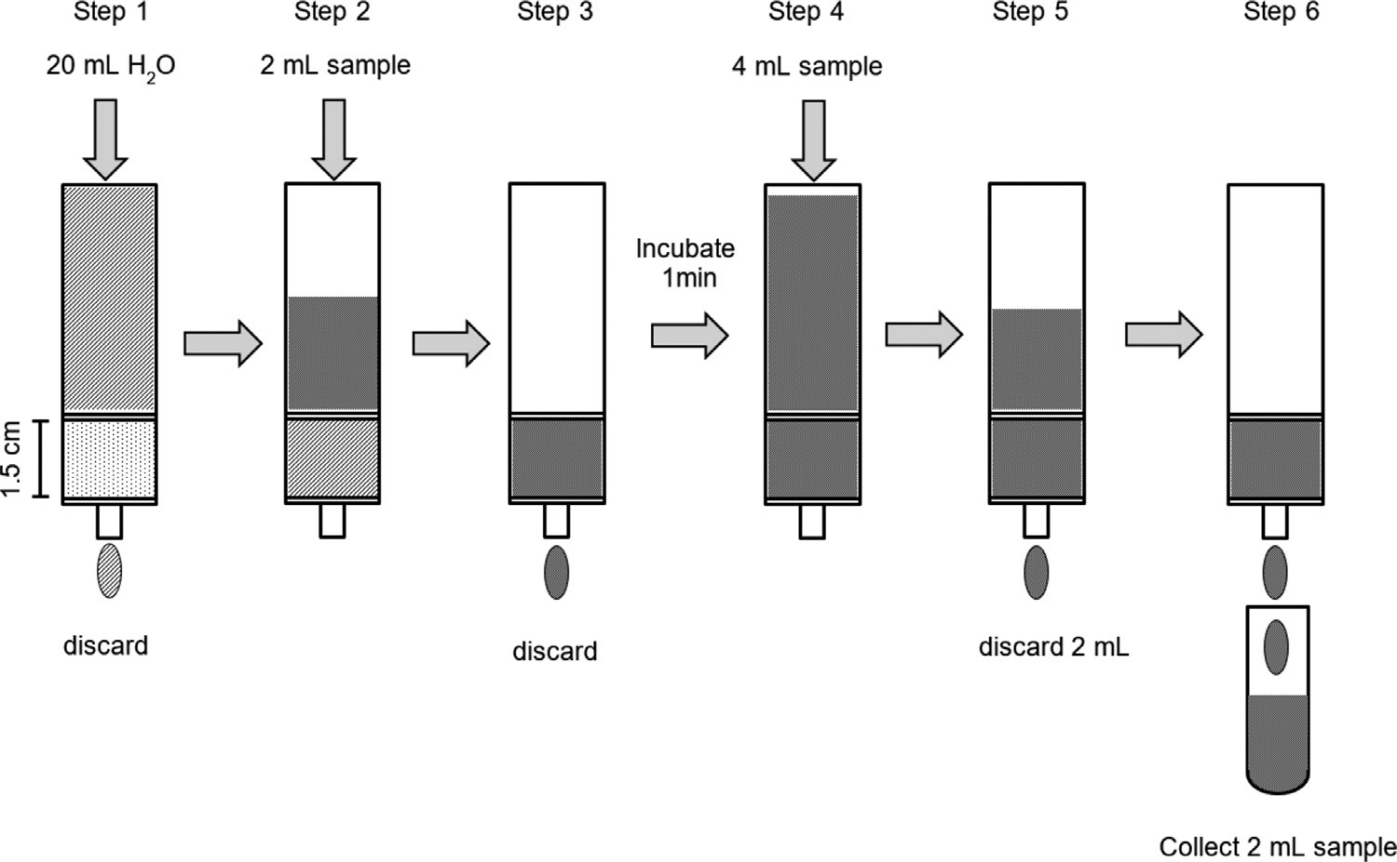
Scheme of the optimised sample clean-up procedure with Dowex 50WX8.

The complete procedure is illustrated below (Fig. 3).

1. Set 6 mL bond elution reservoir in vacuum filtration unit
2. Fit reservoir with frit at the bottom
3. Fill up the reservoir with homogenised wet Dowex 50WX8 corresponding to 1.75 g wet resin material
4. Cover resin with frit
5. Rinse CER bed with 20 mL UPW (step 1)
6. Add 2 mL standard sample solution containing phosphonates such as AMPA (step 2)
7. Elute with flow rate of 1 mL min^−1^ standard solution until the CER column bed is still covered
8. Discard eluent (app. 2 mL)
9. Incubate CER bed for 1 min (step 3) to prevent AMPA exchange against hydrogen ions during the final clean-up step
10. Add 4 mL standard solution on the CER bed (step 4)
11. Elute standard solution and discard the initial 2 mL (step 5)
12. Continue to elute the standard solution and collect the final 2 mL (step 6)

## Method validation

### Quality control

**Fig. 4:**
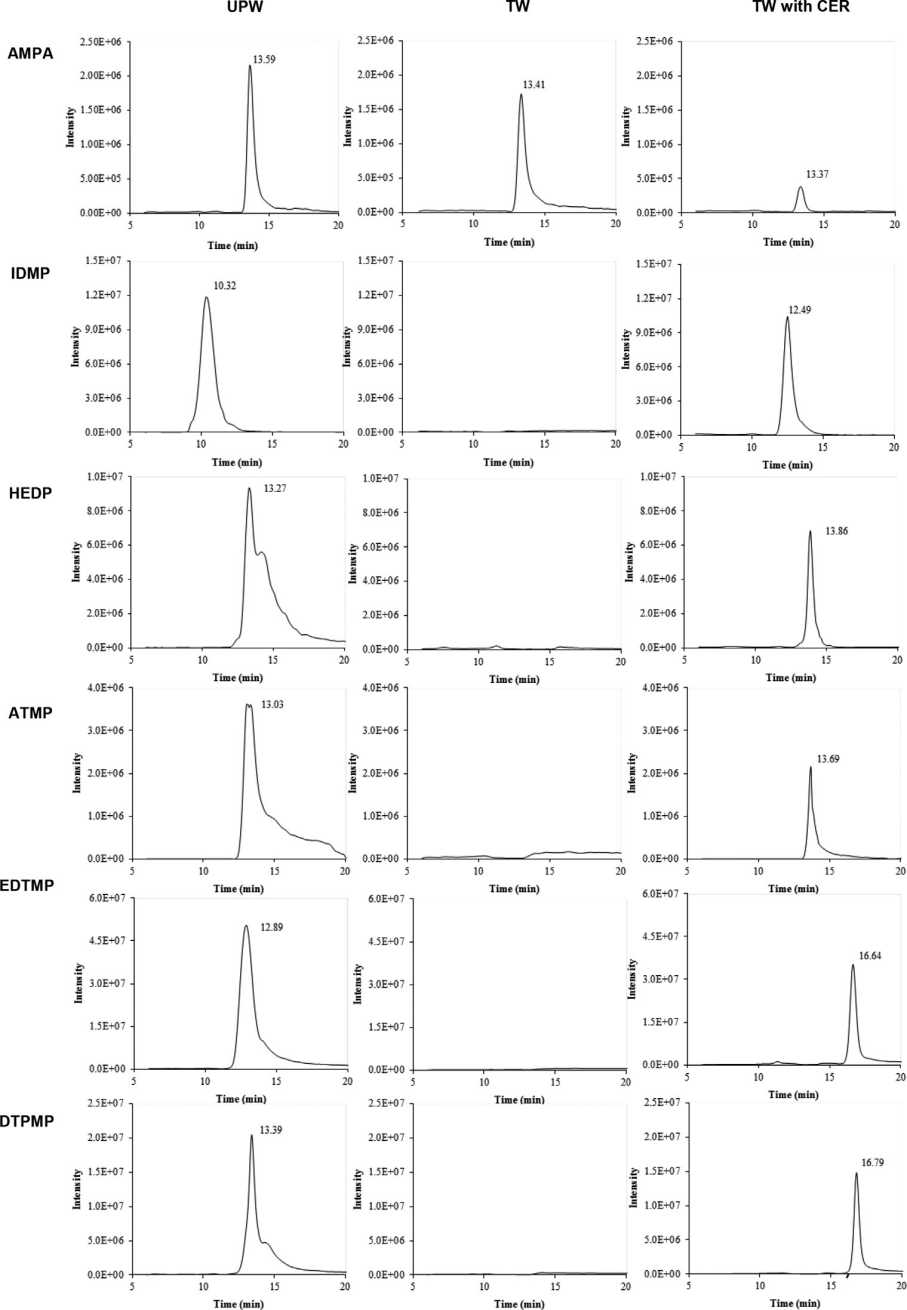
Qualitative control of the sample clean-up procedure of the six standard phosphonates (each 5 mg L^−1^) via LC/MS analysis.

The suitability of CER Dowex 50WX8 as a simple and fast sample clean-up procedure for phosphonates was tested first with the conventional clean-up procedure. Standard solution with two different water qualities (UPW and TW) were qualitatively analysed by LC/MS (Fig. 4).

Phosphonates dissolved in UPW (5 mg L^−1^ each) can be easily analysed with LC/MS without derivatisation (Fig. 3). At least AMPA, IDMP and EDTMP show peaks with an acceptable resolution and individual retention times (RT). The three phosphonates HEDP, ATMP and DTPMP indicate slight tailing most probably due to inhibited retention and/or other separation problems caused on the HILIC LC column.

Only AMPA is then detectable by LC/MS analysis in case phosphonates are prepared in TW (5 mg L^−1^ each) and no purification with the clean-up procedure is performed prior. However, the recovery of AMPA also significantly decreased. The other five standard phosphonates are not detectable as single peaks.

In contrast, phosphonates dissolved in TW (5 mg L^−1^ each) and purified with the clean-up procedure show single peaks for all analysed phosphonates with excellent peak resolution except for AMPA. The latter is still detectable but the recovery is decreased significantly. A shift in the RTs occurred for all six phosphonates compared with the standard phosphonates in UPW. This might be attributed to different ion concentrations affecting the LC-behaviour as recently demonstrated by Skeff et al. for the three phosphonates glufosinate, AMPA and 2-aminoethylphosphonic acid (2-AEP) [6].

### The influence of incubation time and addition of CH_3_COONH_4_

**Fig. 5:**
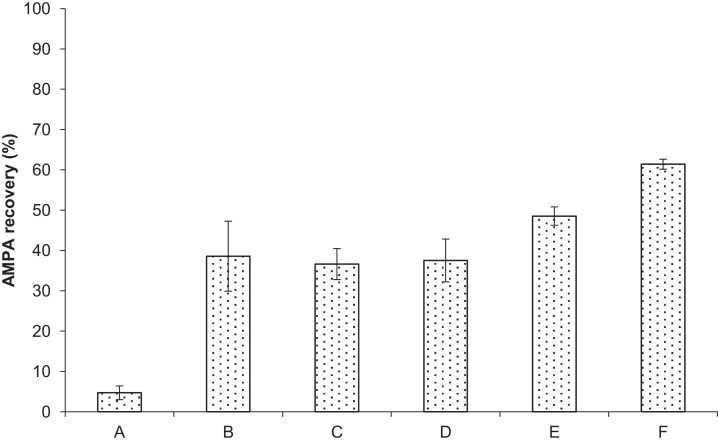
Influence of incubation time and addition of different concentrations of CH_3_COONH_4_ for successful AMPA recovery_._ The 10 mg mL^−1^ AMPA standard solution was prepared with UPW. A: 0 min without CH_3_COONH_4_, B: 0 min with 100 mg L^−1^ CH_3_COONH_4_, C: 0 min with 1000 mg L^−1^ CH_3_COONH_4_, D: 1 min without CH_3_COONH_4_, E: 1 min with 100 mg L^−1^ CH_3_COONH_4_, F: 1 min with 1000 mg L^−1^ CH_3_COONH_4_.

The incubation time on the CER can have an important influence on the resulting sample purity, especially, if the phosphonate AMPA is the target being cleaned-up. In presence of protons, AMPA protonates easily and retains on the resin (Fig. 5, condition A). The recovery of AMPA will automatically decrease in purified samples. This obstacle can be minimised by including, for example, an incubation step of 1 min. This time is enough to saturate the resin with surplus AMPA and will lead to prevent further AMPA exchange against hydrogen ions during the final clean-up step (Fig. 5, condition D). Similar effects to increase the AMPA recovery can be obtained by increasing the counter ion concentration like ammonium (Fig. 5, condition B & C). Since the LC/MS analysis is running with CH_3_COONH_4_ in the gradient eluent the use of the same salt is favoured. Other salts such as ammonium formate can be also utilized where also ammonium is the counter ions. The use of sodium acetate or potassium acetate might also be applicable and should be additionally proven. Interestingly, combing both influence parameters further improves the recovery of AMPA (Fig. 5; E & F). Thus, 1 min incubation on the resin and higher counter ion concentrations (i.e. 1000 mg L^−1^ CH_3_COONH_4_) lead to increased AMPA recovery up to 60 %.

### The influence of the pH

**Fig. 6:**
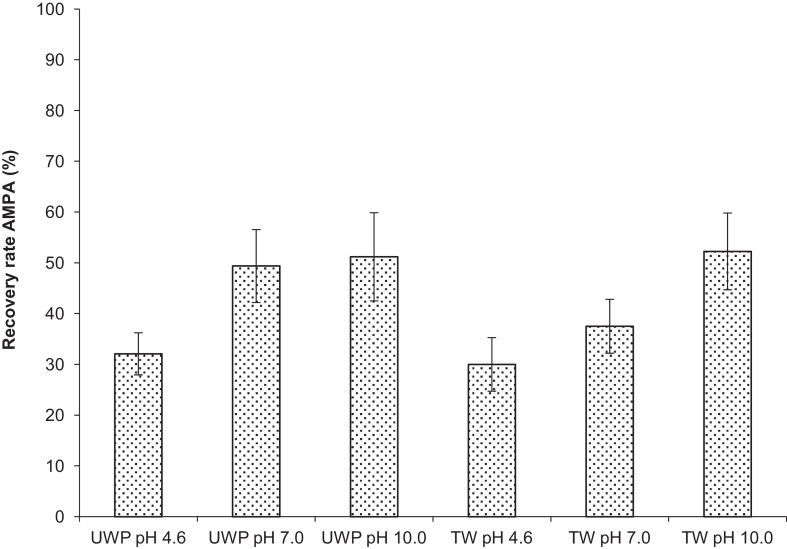
Influence of the pH on the recovery of 10 mg L^−1^ AMPA without additional 1000 mg L^−1^ CH_3_COONH_4_.

The influence of the pH on the sample before the clean-up with CER is of major importance. Acidic samples provide surplus protons decreasing the ion exchange on the resin (Fig. 6). Almost similar recoveries about 30 % are obtained for both AMPA standard solutions (i.e. UPW &TW) and achieved about 30 % only. AMPA samples in UPW with neutral pH result in higher recovery compared with those in TW. The AMPA sample in UPW provides less cations to exchange on the resin minimising the pH gradient that occurs on the resin for TW samples. The latter contains higher concentration of cations such as calcium, magnesium and sodium that also exchange on the resin during the clean-up. Especially during the sample incubation of 1 min the liquid phase on the resin increases the hydrogen concentration and decreases the pH. Slight decreases of the pH are enough progressing the protonation of AMPA which results in strong adsorption on the resin and loss in recovery. Thus, a higher pH of the sample before the clean-up prevents proton saturation during the incubation time on the resin and result for both water qualities, i.e. UPW and TW, in higher recoveries up to 52.3 %. At higher pH value above 9.0 CH_3_COONH_4_ is not usable as counter ion since ammonia gas might be released.

### Suitability of the optimised sample clean-up with different phosphonates

The optimised sample clean-up procedure was further validated for their recoveries for all six phosphonates. The individual reference samples are the corresponding standard phosphonate without sample clean-up (Tab. 1 & 2). The limit of detection (LOD), limit of quantification (LOQ), lower limit of quantification (LLOQ) were determined based on the standard deviation and the slope of the calibration curves [7], [8], [9]. The imprecision of the method was determined by calculating the absolute and relative process standard deviation (PSD). The recovery values were calculated by comparing the analytical response for the untreated standard phosphonate with the treated phosphonate.

**Table 1:** Validation of the sample clean-up procedure of AMPA, IDMP and HEDP

Phosphonate	Condition	LOD mg L^−1^	LLOQ mg L^−1^	PSD-	rel. PSD %	Slope-	Recovery %
AMPA (UPW)	Without CER	0.241	1.262	0.098	1.395	0.1584 ± 0.0028	-
	CER	2.237	3.417	0.192	3.197	0.0590 ± 0.0025	37.2 ± 1.59
	CER 100	3.588	4.704	0.093	1.161	0.0864 ± 0.0036	54.5 ± 2.28
	CER 1000	0.619	1.941	0.132	1.917	0.0516 ± 0.0040	32.6 ± 2.55
AMPA (TW)	Without CER	0.530	2.943	0.122	2.041	0.0539 ± 0.0019	34.0 ± 1.18
	CER	0.864	2.392	0.166	2.376	0.0629 ± 0.0020	39.7 ± 1.27
	CER 100	0.476	2.330	0.154	2.372	0.0768 ± 0.0032	48.5 ± 2.03
IDMP (UPW)	CER 1000	1.072	1.926	0.100	1.424	0.0841 ± 0.0025	53.1 ± 1.59
	No CER	0.618	1.643	0.133	2.042	0.9002 ± 0.0214	-
	CER	0.517	0.854	0.151	2.329	0.4410 ± 0.0300	49.0 ± 3.34
	CER 100	1.767	2.633	0.125	1.786	0.4929 ± 0.0216	54.8 ± 2.40
	CER 1000	1.748	2.624	0.089	1.271	0.5254 ± 0.0153	58.4 ± 1.70
IDMP (TW)	CER	1.612	2.328	0.058	0.860	0.5727 ± 0.0175	63.6 ± 1.95
	CER 100	1.966	2.601	0.123	1.885	0.5536 ± 0.0233	61.5 ± 2.59
	CER 1000	1.603	2.157	0.065	0.924	0.5141 ± 0.0208	57.1 ± 2.31
HEDP (UPW)	No CER	4.971	5.192	0.176	2.353	0.7207 ± 0.0174	-
	CER	4.944	5.237	0.148	1.639	0.7033 ± 0.0168	97.6 ± 2.33
	CER 100	4.500	4.705	0.107	1.193	0.4538 ± 0.0194	63.5 ± 2.70
	CER 1000	3.284	3.858	0.100	1.250	0.3508 ± 0.0175	48.7 ± 2.42
HEDP (TW)	CER	3.233	4.028	0.122	1.521	0.5192 ± 0.0210	72.0 ± 2.91
	CER 100	3.904	3.450	0.327	4.085	0.3737 ± 0.0156	52.1 ± 2.17
	CER 1000	1.142	2.728	0.228	3.257	0.2333 ± 0.0144	31.4 ± 3.13

**Table 2:** Validation of the sample clean-up procedure of ATMP, EDTMP and DTPMP

Phosphonate	Condition	LOD mg L^−1^	LLOQ mg L^−1^	PSD -	rel. PSD%	Slope-	Recovery%
ATMP (UPW)	Without CER	1.281	1.901	0.150	2.498	0.3590 ± 0.0120	-
	CER	5.214	5.845	0.221	2.606	0.3473 ± 0.0162	96.7 ± 4.51
	CER 100	3.119	4.479	0.145	1.936	0.3854 ± 0.0153	> 100
	CER 1000	1.404	2.796	0.083	1.179	0.3969 ± 0.0109	> 100
ATMP (TW)	CER	0.586	1.873	0.111	1.818	0.1877 ± 0.0113	52.5 ± 3.14
	CER 100	0.559	2.427	0.109	1.809	0.2109 ± 0.0100	58.8 ± 2.78
	CER 1000	1.367	1.447	0.142	2.029	0.2142 ± 0.0111	59.7 ± 3.09
EDTMP (UPW)	No CER	6.045	11.615	0.196	0.981	0.4341 ± 0.0126	-
	CER	14.348	15.286	1.067	5.335	0.4230 ± 0.0098	97.5 ± 2.26
	CER 100	11.827	13.880	0.663	3.157	0.2329 ± 0.0076	53.6 ± 1.76
	CER 1000	5.706	8.093	0.416	2.082	0.2177 ± 0.0165	50.2 ± 3.81
EDTMP (TW)	CER	5.870	8.741	0.253	1.267	0.3301 ± 0.0146	76.1 ± 3.36
	CER 100	8.623	12.340	0.198	1.040	0.4926 ± 0.0164	> 100
	CER 1000	6.598	12.787	0.437	2.300	0.4280 ± 0.0167	98.6 ± 3.86
DTPMP (UPW)	No CER	9.913	12.703	0.252	1.677	0.4700 ± 0.0123	-
	CER	10.195	14.242	0.160	1.144	0.2495 ± 0.0079	53.1 ± 1.68
	CER 100	8.690	10.595	0.123	0.818	0.1876 ± 0.0099	39.9 ± 2.10
	CER 1000	9.067	10.349	0.143	0.952	0.3896 ± 0.0161	82.9 ± 3.42
DTPMP (TW)	CER	8.372	11.719	0.202	1.348	0.3411 ± 0.0119	72.6 ± 2.53
	CER 100	8.872	12.630	0.190	1.266	0.3176 ± 0.0165	67.6 ± 3.50
	CER 1000	8.951	13.554	0.185	1.233	0.2626 ± 0.0113	55.9 ± 2.41

The different phosphonates result in different preference clean-up conditions, which are mainly caused by the different possible chemical interactions with Dowex 50WX8. The sample clean-up procedure in UWP with addition of 1000 mg L^−1^ CH_3_COONH_4_ result in the highest recoveries for AMPA, IDMP, ATMP and DTPMP. For HEDP and EDTMP, the recoveries decrease by increasing the CH_3_COONH_4_ concentration. However, the recovery value alone is not as essential as LLOQ, LOD and precision for methods validation, therefore, has to be included. Comparing those parameters, the addition of 1000 mg L^−1^ CH_3_COONH_4_ is recommended for AMPA, HEDP, ATMP, EDTMP and DTPMP for the sample clean-up. The phosphonate IDMP alone does not necessarily require the addition of CH_3_COONH_4_ for the clean-up. In case, the sample analysis is combined with other phosphonates as target compounds the addition of CH_3_COONH_4_ is applicable and result is acceptable recovery.

In TW, the phosphonates AMPA, IDMP, ATMP and EDTMP showed highest recoveries with the addition of 1000 mg L^−1^ CH_3_COONH_4_. For HEDP and DTPMP the highest recovery is determined without CH_3_COONH_4_. Considering also LOD, LLOQ and PSD, the sample clean-up with 1000 mg L^−1^ CH_3_COONH_4_ is suitable for all tested phosphonates except DTPMP. For this phosphonate no addition of CH_3_COONH_4_ might be favoured. The individual recommended condition of all six phosphonates tested in UPW and TW is summarized below (Tab. 3).

**Table 3:** Recommended individual sample clean-up condition.

	Treatment	AMPA	IDMP	HEDP	ATMP	EDTMP	DTPMP
UPW	without CER	+ + +	+ + +	+ + +	+ + +	+ + +	+ +
	with CER	+ +	+ + +	+ +	+ +	+	+ +
	CER 100	+ +	+	+ +	+ +	+ + +	+ +
	CER 1000	+ + +	+	+ + +	+ + +	+ + +	+ + +
TW	without CER	+	-	-	-	-	-
	with CER	+	+ + +	+ +	+ +	+ +	+ + +
	CER 100	+ +	+ +	+	+ + +	+ + +	+ +
	CER 1000	+ + +	+ +	+ + +	+ +	+ +	+

Criteria of Table S3:

+++ lowest LLOQ, lowest LOD, lowest PSD, highest recovery value

++ low LLOQ, low LOD, low PSD, high or middle recovery value

+ relatively low LLOQ and/or LOQ, low PSD, fairly recovery value

- no peak detection possible

## Declaration of Competing Interest

The authors declare no conflict of interests.
